# A Review on the Obtaining of Functional Beers by Addition of Non-Cereal Adjuncts Rich in Antioxidant Compounds

**DOI:** 10.3390/antiox10091332

**Published:** 2021-08-24

**Authors:** Rodrigo A. M. Paiva, Yhan S. Mutz, Carlos A. Conte-Junior

**Affiliations:** 1 Center for Food Analysis, Technological Development Support Laboratory (LADETEC), Avenida Horácio Macedo 1281, Polo de Química, Bloco C, Ilha do Fundão, Cidade Universitária, Rio de Janeiro 21941-598, Brazil; rodrigomonroe@outlook.com; 2 Graduate Program in Food Science (PPGCAL), Institute of Chemistry (IQ), Federal University of Rio de Janeiro (UFRJ), Cidade Universitária, Rio de Janeiro 21941-909, Brazil

**Keywords:** bioactive beer, total phenolic compounds, antioxidant capacity, sensorial profile, health benefits, bioavailability

## Abstract

Beer is one of the oldest and most consumed beverages worldwide, and recent trends point to increased consumption of functional beers. However, there is a lack in the scientific literature on the effects of adding functional adjuncts in distinct steps of the manufacturing process and its implications on the final physicochemical and sensorial profile. Therefore, the present review analyzes the ingredients used and their insertion stage to achieve a functional beer with bioactive compounds, higher antioxidant activity, and improved sensory characteristics. The addition of fruits, herbal extracts, plants, and mushrooms in beers was documented. Furthermore, adjuncts were successfully added in wort boiling, fermentation, maturation, and packaging. The wort boiling step stands out among these four due to the superior extraction of phenolic compounds from the added adjuncts. On the other hand, adjunct addition in the maturation step induced low increases in antioxidant and phenolic content of the respective enriched beers. Fruits represented the majority of adopted adjuncts among the studies evaluated. Furthermore, the addition of fruits represented a positive increment in the beer’s volatile profile and an increase in sensory acceptability. A gap in the literature was found regarding the analysis of phenolic compounds with appropriate techniques such as HPLC-MS. Furthermore, there is a need to study the bioavailability of the incorporated bioactive compounds to prove the health claims inferred about these beers. In conclusion, functional beers are a little-explored relevant field, with potential for new studies.

## 1. Introduction

One of the definitions of beer is part of the Code of Federal Regulations (CRF) of the United States of America. According to 27 CRF § 25.11, the term beer means “beer, ale, porter, stout, and other similar fermented beverages (including sake or similar products) of any name or description containing one-half of one percent or more of alcohol by volume, brewed or produced from malt, wholly or in part, or from any substitute therefor” [1]. This definition is comprehensive and is directly related to the conventional beers vastly marketed. Beer is the most consumed alcoholic beverage globally, with remarkable growth in China, Brazil, and India [2,3].

Despite being the most consumed alcoholic beverage worldwide, beer is a subject of constant research [4]. The research topics address the improvement of several characteristics, such as foam formation stability [5], beer aging [6], and research and development of non-traditional beers [7,8]. Among non-traditional beers, functional beers stand out, as they seek to combine moderate consumption of the drink with health benefits [9]. This perspective aligns with the new market trend of functional beverages that attract consumers based on their perception of disease prevention through a functional diet [10]. The moderate consumption of beer is already of health interest as the beverage is rich in amino acids, minerals, vitamins, and phenolic compounds [9]. Furthermore, the insertion of non-amylaceous adjuncts to beer seeks to provide the release of bioactive molecules, such as those with antioxidant and nutraceutical capacities, besides contributing to the final sensory profile of the product [11].

Even though the act of brewing is millenary, its biochemical principles remain the same [12]. Therefore, the challenge is to add new compounds such as fruits, herbs, and spices, without impairing the already established manufacturing process. As distinct adjuncts are available and several manufacturing steps can be adequate for the adjunct addition, a new functional beer design requires a detailed study on the physicochemical and sensory variables of the process. However, few articles comprehensively study the addition of adjuncts and how their classification, addition mechanism, and insertion stage influence the final beer’s chemical, physicochemical, and sensorial characteristics.

Another gap in the literature points to the sensory analysis of the developed product, which is not unanimous in the studies, which is a severe issue once the sensory analysis is an imperative tool to evaluate the acceptance of novel beers by potential consumers and move forward with the project [6]. In this sense, the present review aims to evaluate the actual state of the functional beers from production approaches to the achieved chemical, physicochemical and sensorial attributes and resulting content of bioactive molecules and antioxidant activity.

## 2. Potential Production Steps and Addiction Mechanism for Inclusion of Brewing Adjuncts

Traditional beer produced with water, malted barley, hops, and yeast is naturally a complex matrix composed of several compounds [13]. Nonetheless, it has a well-established manufacturing process. Therefore, making a non-traditional beer involves balancing this contrast between complexity and robustness, which requires technical knowledge from the producer in the creation process [14]. Furthermore, another challenge is the conjoint analysis of the insertion step and the adjunct composition and form (dehydrated, in pieces, extract, pulp, or microencapsulated) [15]. However, as each manufacturing step has its control variables and requires technical and empirical knowledge, it becomes possible to filter a suitable set of options and simplify the number of potential combinations to be tested.

Therefore, it is essential to feed the process with suitable adjuncts that can meet the needs of each unit operation, focusing on the characteristics of the desired product. Non-traditional beers can be separated into five classes: low-calorie beers, low-alcohol or alcohol-free beers, beers with a new flavor, gluten-free beers, and functional beers [7]. Functional beverages are a trend in the academic and industrial environment because they are among the new frontiers in food science, which can be seen from the growing number of publications involving new ingredients, production processes, and health benefits [10].

### 2.1. Adjunct Addition in Wort Boiling

By directly analyzing the production flowchart, it is possible to identify potential stages of the brewery eligible to provide benefits when adjuncts are inserted. The first manufacturing step that meets the selection criteria is wort boiling, as it is intertwined with a crucial intensive thermodynamic property: temperature. The traditional brewing process alone is abundant in several chemical compounds. Naturally, from the boiling process, a mixture of acids, alcohols, aldehydes, esters, furans, hydrocarbons, ketones, lactones, pyrazines, phenol, and sulfur-containing already emerge [13]. Moreover, adding novel compounds to produce a functional beer will increase such sensorial complexity. On that aspect, the thermal load applied in the boiling step simultaneously influences two antagonistic aspects: the evolution of volatile compounds and the extraction of bioactive compounds [16].

Xu et al., pored over these facets and analyzed the consequences of adding okra pulp and dried okra on a cloudy wheat beer [17]. Okra possesses beneficial compounds such as vitamins, minerals, and conjugated linoleic acid (CLA). In addition, okra is rich in water-soluble carbohydrates, especially pectin, responsible for increasing wheat beer viscosity and providing a higher yeast suspension, an intrinsic characteristic of wheat beers. Through sensory analysis, it was found that the presence of okra in the formulation increased the beers’ turbidity, foam smoothness, and stability. The beer made with fresh okra presented a higher concentration of terpenes such as styrene, typical in the flavor of cloudy wheat beer. Moreover, the fresh okra beer was also the only beer to present caryophyllene, thus presenting a new aroma.

Although healthy, the addition of conjugated linoleic acid (CLA) concerns the sensory quality of the beer. Linoleic acid is a polyunsaturated fatty acid known as omega-6 and found in abundance in oil extracted from okra seeds. Although researches show that it is necessary to increase the intake of CLA in the daily diet and highlight its anti-inflammatory and anti-carcinogenic potential [18], from a technological perspective, the oxidation of this set of fatty acids generates off-flavor compounds, such as trans-2-nonenal [13]. In beer, this unsaturated aldehyde confers an unpleasant cardboard flavor, long known in beer sensory studies [19]. Overall, good results have been obtained with the addition of okra in the formulation of cloudy wheat beer. The sensory profile and aroma of the new beer were improved, and no off-flavors were found. In light of being abundant in antioxidants, okra aggregates positive health appealing to the product. In their review, Gemede, Ratta, and Haki describe the composition of okra and strengthened the recommendation to add okra to different formulations due to its health benefits [20].

In their work, Deng et al., added dried magnolia berries (also known as omija fruit) to an ale-type beer and evaluated the effects on its composition and sensory characteristics [11]. In line with the previous study’s findings, the association of a fruit adjunct and the boiling step was successful. Omija fruit was also added in steps after boiling. However, the addition of the fruit after boiling leads to a lower extraction of phenolic compounds and reduced sensory attributes and oxidative stability. Adopting omija as an adjunct is also valuable as it is rich in lignans, which are bioactive compounds. In omija berries, they are mainly present in the peel and seeds [21]. Therefore, they become more accessible when dried berries are crushed before use, as performed in the present study [11].

Three main lignans (schisandrin, gomisin A, and gomisin B), absent in control beer, were detected in omija-enriched beers, with the higher lignans content being achieved when berries were added to the wort boiling. As the authors pointed out, the boiling process conditions do not degrade lignans; on the contrary, they promote greater extraction. On the other hand, omija fruits are also rich in anthocyanins, and their extraction demands attention as their addition to the boiling process increased the beer’s color (EBC). However, the color increase did not affect the total acceptability of the produced beer. Unfortunately, the beer’s color attribute was not individually analyzed during the sensory evaluation. Therefore, it is inconclusive whether this fact alone had a positive or negative weight in the final perception, and care should be taken when adding coloring adjuncts to beer.

Another example of good results was obtained with dried goji berry (also known as wolfberry), a fruit originally from Asia. Ducruet et al. prepared a beer enriched in bioactive compounds and with better sensorial characteristics when the berries were added during wort boiling [22]. In this case, when not added at the beginning of the brewing process, the results were not equally satisfactory. The analysis of beer color intensity showed that beers with higher color intensity were those produced with the addition of ground goji berry in the boiling stage, reflecting a higher extraction of berry compounds due to the temperature and higher surface area.

It was concluded that independent of the addition step, the addition of 50 g/L of goji berries increased the antioxidant capacity of the final beer. However, higher phenolic and antioxidant content were obtained for the addition in the initial stages of production. One class of compounds contributing to the observed increase in antioxidant levels other than phenolics is the carotenoids, natural dyes that give an orange tone to the berries [23].

The incorporation of wolfberry in early production steps promoted the reduction of beer turbidity due to the precipitation of proteins that potentially interact with polyphenols. On the other hand, addition right before bottling leads to higher turbidity due to the suspension of fruit particles not being removed on filtration. These findings highlight the success of the dual fruit-wort boiling approach when it comes to enriching a beer.

### 2.2. Adjunct Addition in Fermentation

The next manufacturing step in the sequence, fermentation, is also a candidate for producing a functional beer due to its technological flexibility. On the one hand, this step’s flexibility derives from the participation of a living organism with several active metabolic pathways [24]. On the other hand, this same feature adds complexity to this production step. Although the biochemistry of the fermentation itself is well established, recent advances in the integration between genomics, transcriptomics, and metabolomics of beer yeasts have unraveled in detail its main metabolic routes [25]. Moreover, genetic engineering allows the manipulation of genetic material to improve the production of certain metabolites. Aromatic amino acid metabolic pathways, for example, are cited as candidates for enhancement through genetic manipulation of *Saccharomyces cerevisiae*, as it is possible to produce flavonoids and stilbenes from glucose via aromatic amino acid precursors [26]. Therefore, the fermentation step offers a double opportunity to obtain bioactive compounds: directly from the adjuncts themselves or from the metabolization of compounds aggregated with adjuncts.

Cho et al., investigated the direct effects of adding persimmon fruit in the fermentation stage [27]. This approach can be considered an initial feasibility study for applying fruit at this stage. They examined a range of concentrations between 50–200 g of fruit per 10 L of water and how the fruit impacts brewed the resulting beers’ quality characteristics and antioxidant activity. The choice of this fruit encompassed both the rising local productive potential and nutritional aspects of interest in the fruit.

The tests included pH, titratable acidity, alcohol concentration, instrumental color, total phenolic content, and antioxidant capacity. A significant increase in pH and titratable acidity were attributed to the fruit’s high pH and production of organic acids, respectively. However, no changes in alcohol content were found. Such a result is positive, as one of the obstacles in adding fruits in the fermentation step is the possible additional alcoholic fermentation from the fruit sugar. However, it is recommended that studies in this area include storage experiments and post-production analyses to assess the effects of these pH variations as they can negatively affect the product during storage. Indeed, is reported in the literature that lower pH values lead to greater oxidation of isohumolones and less flavor stability during storage [28].

The viability of adjuncts addition in fermentation is corroborated by Nardini and Garaguso. In their work, the researchers characterized the bioactive compounds and determined the antioxidant activity of commercial fruit-beers [29]. In detail, raspberry was added to a lambic-style beer, while fresh pieces of cherry, peach, apricot, grape, plum, orange, and apple were added to ale-style beers. Orange beer stood out, in comparison with others, displaying the second-highest value of total polyphenols, despite being the beer that utilized the lowest amount of fruit (0.5% *w*/*v*) compared with the other fruit beers analyzed. This result was achieved because orange peel is richer in phenolics than its pulp. Orange peel has an elevated nutritional value and contains several antioxidant compounds, including ascorbic acid, flavonoids, and pectins [30]. Indeed, different parts of the fruit can be used and should be tested as their composition affects the final incorporated bioactive molecules differently.

The total polyphenols content was influenced by the amount of fruit used and the beer style adopted [29]. The influence of beer style becomes apparent when comparing lambic and ale raspberry beers. Although the ale style received three times more raspberry than the lambic counterpart, it showed a lower total polyphenols content value. On the other hand, the higher amount of fruit added to the ale style raised the antioxidant capacity and total flavonoid content to values higher than the lambic style.

The fermentative performance also requires special attention when higher percentages of fruits are supplemented to the sweet wort. Nunes et al., and Melo et al., related the addition of 30% of cocoa pulp and 29% of bush passion fruit pulp, respectively, as a complement to malted barley [31,32]. This procedure was accompanied by a temporal evaluation of the following common fermentative parameters: cell growth, substrate consumption (glucose, fructose, maltose, and maltotriose), and ethanol production. Analysis of these three indices allows for the follow-up of the fermentative efficiency and control. In addition to the genetic characteristics of yeast, it is known that a high initial load of fermentable sugars and the concentration of ethanol are primary inhibitors of yeast growth [33]. While Melo et al., evaluated different concentrations (10%, 29%, 39%, and 49%) of bush passion fruit pulp, Nunes et al., investigated a single concentration applied to two strains of *S. cerevisiae* (SC52 and S-04). While the former found the highest ethanol production with 39% pulp, the latter indicated SC52 strain as the one that responded best in substrate consumption and ethanol production with a high viability percentage. These represent two viable ways to produce fruit beers, the control of fruit content or the selection of specialized yeast strains. In addition, banana juice used by Carvalho et al. also proved to be viable as a partial substitute for an all-malt wort. The group recorded volumetric productivity in ethanol of approximately 0.60 g/L·h [34]. This value is close to that obtained by Nunes et al. after 84 h of fermentation with both strains.

### 2.3. Adjunct Addition in Maturation

The act of transforming beer into a beverage with higher health benefits also involves the assistance of maturation. At this stage, the constant and intimate contact between adjuncts and beer results in refined flavors due to the solubilization of bioactive compounds and reactions that allow the equalization of off-flavors, such as aldehydes, vicinal diketones and sulphur compounds [35]. Several raw materials, historically known for their benefits, can be combined with an alcoholic beverage during its manufacturing. Therefore, it is essential to explore local products and typical ingredients and aggregate knowledge from other areas such as herbalism to gather information and scientific methods to allow its use [36].

Aiming to exploit these positive points of local products, Bustos et al. added dry leaves of *Parastrephia lucida*, a plant originally from South America, to a porter craft beer for 24 h at the beginning of its maturation [37]. After the contact period, leaves were removed, and the maturation proceeded for another six days. The final product was a beer enriched in phenolic compounds and with higher antioxidant capacity. However, the final beer alcohol, color bitterness, and standard technological parameters were retained. The researchers emphasize that adding plant material at this stage was preferable because the maximum intensity of the phenolic content can be recovered. Further, clarification and filtration are not recommended after the maturation to obtain a higher content of bioactive compounds, as the addition of flocculants such as polyvinylpolypyrrolidone (PVPP) could reduce these substances’ presence in the final beer.

Adadi et al., examined sensorial acceptance for a Kölch beer added with sea buckthorn berry [38]. The fruit, typical of northwestern Europe and central Asia, was added in mashed form and mixed into the beer at the maturation stage for two weeks, after a four-week fermentation. The new beer’s aroma was sensory evaluated with a higher average score than the control beer. Moreover, the beer produced with buckthorn berries had greater antioxidant capacity than the other two commercial beers analyzed by the authors.

In addition to fruits and plants, other natural ingredients are qualified to be an adjunct in the brewery industry, with propolis being one of them. Propolis is naturally rich in biologically active substances, especially antioxidant molecules as phenolic compounds [39,40]. This attribute has particular technological importance since it allows extending the shelf life of the product. Furthermore, the adoption of propolis in beverages and foods should be highlighted for the role of its phenolic compounds in disease prevention and immune system boosting [41]. The total maturation time for the beer produced by Ulloa et al., was 24 days, with a 10-day cold maturation and a 14-day maturation at room temperature. The propolis extract was added after two days of cold maturation after stabilization of beer by PVPP. The addition of extract after stabilization allowed for a higher content of bioactive compounds that could be lost if added before a clarification process. Technologically, propolis extract is also useful as an enzyme inhibitor and should be explored to increase the shelf life of this beer in future studies.

### 2.4. Adjunct Addition after Packaging

Finally, the literature also describes that brewing adjuncts could be mixed with the beverage after packaging. Unlike the previous steps, there is now the possibility of effective consumer participation in carrying out this task. This, therefore, expands the dimension of the functional beer market since it becomes possible to add different compounds and personalize the final drink, also impacting the consumer experience. In this scenario, aseptic care is of utmost importance. In their work Vaughan et al. emphasized the impacts of inefficient asepsis during the brewing process [42]. The authors divided the sources of contamination into primary contamination and secondary contamination. While in the first, contaminating agents come from raw materials and production equipment, in the second, these agents end up being added during packaging. Thus, it is possible to infer that both can occur during the normal processes of adding adjuncts. Moreover, the after- packaging addition can result in a loss of carbon dioxide as the finished product needs to be opened.

Aligned with the described scenario, Djordjevic et al., tested a diverse group of adjuncts in the form of plant extracts: *Melissae folium*, *Thymi herba*, *Urticae radix*, *Juniperi fructus*, and *Lupuli strobuli* [43]. Extracts were carefully obtained based on the pharmacopeia and aseptically added to a commercial pilsner beer. The extracts were obtained by a single-percolation process, and the final ratio of plant material to solvent (ethanol-water extract) of 1:2 was achieved. The research group carried out a preliminary study of the added extract concentrations to optimize the beer consumer acceptance and functional properties. The amount was between 0.45–0.70 mL of extract per liter of beer. After the extract addition, the beers were closed and remained in maturation for days at 10 °C. It should be highlighted that the concentrations used did not exceed the therapeutic dose indicated for each pure extract’s use. Positively, all beers with adjuncts had an increase in phenolic content, reaching 37.09% for the beer with *T. herba* compared to the control beer. Likewise, the antioxidant activity was also increased, and its correlation with the increase in phenolic content was statistically proven.

Beyond plants, roots, fruits, and flowers, mushrooms are emerging beer adjuncts, specifically, the mushroom *Ganoderma lucidum* [44,45]. This traditional Asian medicinal macrofungus has hundreds of distinct secondary metabolites with identified therapeutic potential [46]. Among these compounds, the triterpenoid class, of which 150 molecules have been isolated, is of particular interest [47]. In addition, these macrofungus triterpenoids have documented anti-carcinogenic properties [48]. In general, the benefits found in *G.a lucidum* have increased the number of commercial products that contain it, including functional beers.

In their study, Leskosek-Cukalovic et al., investigated the supplementation of a commercial beer with *G. lucidum* extract. The mushroom extract was added in the range of 0.1–1.5 mL/L of beer, respecting the recommended daily dose of 1.8–4.8 g set by the FDA (FDA, memorandum No 953-03 16, 1999, FDA No 955-0316, 2003) and aiming the sensory acceptability of the product. The maturation was carried out at 5 °C for one day. The sensory evaluation results showed that the *Ganoderma* beer was judged superior to the standard beer in every analyzed attribute by male consumers. In contrast, amongst females, beer with extract was judged inferior in all aspects except the beer body. Furthermore, using liquid chromatography coupled with mass spectroscopy, the authors showed that the triterpenes and fatty acids were successfully incorporated into the beer.

Further topics in methods of incorporating adjuncts after packaging include microencapsulation of bioactive compounds. In this context, Belščak-Cvitanović et al., microencapsulated green tea (*Camellia sinensis* L.) extract and garnea mushroom (*G. lucidum*) extract. Microbeads of pectin and alginate and microcapsules coated with chitosan were obtained by electrostatic extrusion using ionic gelation. The two carrier materials were used for the extracts of mushroom and green tea. Besides, the study included powdered green tea extract obtained by spray dryer equipment. Although they achieved an encapsulation efficiency greater than 50%, it was observed that only dry extracts allowed a significant increase in the total phenolic content. It is worth highlighting the importance of encapsulation technology as microencapsulation and, more recently, nanoencapsulation. However, it may not be efficient, depending on the objective and conduct of the project.

## 3. Evaluation of Bioactive Compounds and Health Benefits

The main objective of evaluating a non-traditional beer classified as functional is to prove its stability as a vehicle for bioactive compounds, which benefit human health. In general, the advantages that adjuncts can bring to the brewing process are concrete [49]. As seen previously, with a scientific methodology, one can merge the possible therapeutic aspects of an adjunct with the fundamentals of process engineering to obtain a functional beer. As a result, when the scope of a traditional beer is extended and functional beers are characterized, specific physicochemical and stability analyses are demanded to corroborate its effectiveness. Within the set of most common tests found in the literature for functional beers are: total phenolic content [29,44,49], phenolic profile [29], antioxidant activity [27,50,51] and volatile profile [17,38,52]. Therefore, to evaluate the performance of the functional beers, the next sections will focus on the phenolic content and antioxidant activity of the beers.

### 3.1. Phenolic Compounds

The presence of phenolics in beer is has long been known in brewing science. Chemically speaking, phenolic compounds are organic aromatic structures with one or more hydroxyl groups linked directly to an aromatic ring, among which phenol is the simplest representative of the class. As organic compounds, phenolic compounds exist in a large number and diversity, so secondary classifications are used to allocate them. The most important classes in beer are flavonoids, phenolic acids, and stilbenes due to their antioxidant characteristics and potential instability and damage to beer quality during prolonged storage, such as permanent hazy from their complexation with proteins [53,54,55].

With a focus on quantifying total phenolic content (TPC), several global tests based on colorimetric methods are detailed [56]. The Folin-Ciocalteu method is very relevant in this field since it is considered technically accessible and not time-consuming. It can be applied in several matrices, facilitating implementation in laboratories [57]. It follows a simple principle based on a colorimetric assay that detects the redox reaction between reducing compounds and the Folin-Ciocaulteu reagent. This reagent is a solution constituted of phosphomolybdic acid, phosphotungstic acid, and lithium salts. After the alkaline medium reaction, the intensity of the blue color from the sample is related to the concentration of reducing substances. The blue tone is characteristic of tungsten oxide and molybdenum oxide, allowing their detection at 760 nm band. The quantification is traditionally made from a standard curve of gallic acid solution [58,59,60]. However, it is essential to emphasize the importance of sample preparation since the method is sensitive to reducing substances and phenolic compounds, which is a limitation of the technique that can cause inconsistencies for samples with complex compositions [57].

The results of TPC for all the analyzed functional beers are shown in Table 1. As it can be seen, fruits represent the major group of adjuncts added in beers. There was no consensus on the addition step, with fruits added on wort boiling, fermentation, maturation, and packaging. Data among different fruits were too distinct to be helpful in comparison. However, when tested among the same fruit, such as for goji berries and omija, the addition in boiling step led to higher TPC values than fruits added in other steps (Table 1). Indeed, according to the review of Martinez-Gomez et al., wort boiling is the most qualified addition step regarding the increase of phenolic compounds in beer within the four possibilities (wort boiling, fermentation, maturation, and packaging) [60]. However, the use of plant extracts showed positive results when added to maturation. Bustos et al., reached a value of 559 mg of gallic acid equivalent per liter (mgGAE·L^−1^) with 10 g·L^−1^ of *P. lucida*. For perspective, Ducruet et al. reached a value of 415 mg GAE·L^−1^ using 50 g·L^−1^ of whole goji berry in the secondary fermentation. Reinforcing the importance of studying the used adjunct to maximize its benefits with less material added. However, an evaluation of clarification is necessary when dealing with adding plants and extracts to maturation. Furthermore, it can be inferred that independent of the addition stage and added adjunct, an increase in TPC can be achieved as long as the process peculiarities are observed.

Phenolic compounds in beer are mainly regarded for their potential health benefits and beneficial antioxidant role in preserving beer flavor. For that reason, the global phenolic content is invariably determined in functional beer studies. However, as mentioned, the phenolic class encompasses many molecules with distinct structures, which implies distinct impacts on the beer and its health properties [53]. Therefore, controversies exist in whether results of TPC indexes are relevant to indicate the bioactive function of these molecules on human health, as the spectrophotometer measure of TPC is considered a screening technique for the phenolic content of food and beverages [63]. To assess the real phenolic content of a matrix, state-of-the-art techniques, such as liquid chromatography-mass spectrometry (HPLC, HPLC-MS) are needed [53,63]. These techniques offer a selectivity not found in the spectrophotometric methods [64].

Furthermore, more specific approaches such as profiling the phenolics present in samples are of much more value regarding the health claims behind a functional beer. Once the health properties of phenolic compounds such as anti-inflammatory and anti-cancer activities are measured individually, on in vivo studies [65,66] such profile descriptions of the phenolics transferred from the adjunct to the final matrix are imperative. However, the number of studies that assess phenolic content and functional beers’ profile using such techniques is low. Only two studies that used HPLC to determine phenolics in beer were found.

Zapata et al. used liquid chromatography with a diode array detector coupled to mass spectrometry using electrospray ionization interface (HPLC-DAD-ESI/MS) to identify the phenolic profile of quince fruit and the profile of the respective enriched beer. The only class of phenolics found were hydroxycinnamic acids. In quince fruit, five such acids were found: neochlorogenic acid, chlorogenic acid, 3-*O*-coumaroylquinic acid, 5-*O*-coumaroylquinic acid, and 3,5-di-*O*-caffeoylquinic acid. However, only chlorogenic acid, neochlorogenic acid and 3,5-di-*O*-caffeoylquinic acid were quantified in the produced quince beers. This class of phenolic acids has described health effects as neuroprotective agents and helping prevent cancer and cardiovascular diseases [67,68]. On the other hand, although hydroxycinnamic acids have a low impact on the beer aroma, their decarboxylation leads to many potent off-flavors.

In their study, Nardini and Garatuso determined the profile of phenolics produced in commercially available fruity beers using HPLC-DAD. In the beer without adjuvants, the authors found exclusively phenolic acids class compounds. Further, vanillic, caffeic, syringic, *p*-coumaric, ferulic, and sinapic acids were all in their conjugated forms. On the other hand, in the fruit beers studied (cherry, raspberry, peach, apricot, grape, plum, orange, and apple), flavonoids as catechin, rutin, myricetin and quercetin, besides resveratrol (a stilbene) were found. In addition, the phenolic acids already found in the beer without fruits were also found in fruity beer in their free form. Flavonoids have been described as having health benefits, including anti-inflammatory activity and cardiovascular protective effects [67]. Therefore, the antioxidant potential of the beers was improved, and the fruit beers can be considered enriched with bioactive compounds.

It is essential to highlight that, even though the putative or even proved in vivo effect of selected phenolic compounds are beneficial to human health, the compound’s bioavailability has to be assessed. The bioavailability represents the proportion of a compound available for utilization after digestion and absorption by an individual [66]. Therefore, as the bioavailability varies between individuals [65], a beverage rich in bioactive molecules cannot be assumed to promote health gains based only on in vitro tests of TPC, antioxidant activity, or HPLC quantification. In this sense, it is worth highlighting a gap in the scientific literature regarding the bioavailability of the bioactive molecules in functional beers.

### 3.2. Antioxidant Activity

From a technological point of view, antioxidants’ role in food matrices has been growing in recent years. Naturally, hops and barley are endogenous sources of antioxidants compounds in beer [4]. According to Bursal et al., the antioxidant potential consists of the ability of a compound to interact with free radicals or non-free radical species and delay or prevent oxidative degradation [69]. Thus, a functional beer rich in antioxidant compounds can be helpful to combat radical species present in the human body in a preventive way. In human health, reactive oxygen species, also known as radical compounds, or free radicals, cause concern once they cause damage to cellular tissues [70]. Free radicals owe their high reactivity to one or more unpaired electrons. Common examples of radicals present in foods are hydroxyl radicals (HO^•^), hydroperoxyl radicals (HOO^•^), and hydrogen peroxide (H_2_O_2_). Although hydrogen peroxide has no unpaired electron, it is capable of general free radicals such as HO^•^. Devasagayam et al. argued that this configuration makes reactive species dangerous in the long run for human health, causing the organism to suffer from oxidative stress [70]. Oxidative stress can be understood as an imbalance between the natural control of reactive compounds triggered by environmental, nutritional, or genetic factors [71].

Thus, to suggest a preventing property against oxidative stress in the body is important for a beer to be enriched with antioxidant compounds [72]. This potential can be evaluated by several colorimetric tests that seek to quantify antioxidant compounds. Colorimetric methods are well evaluated and used for their simplicity and performance. The most commonly applied to beverages are Trolox equivalent antioxidant capacity (TEAC), total peroxyl radical trapping antioxidant parameter (TRAP), 2,2-diphenyl-1-picrylhydrazyl (DPPH), *N*,*N*-dimethyl-*p*-phenylenediamine (DMPD), photochemiluminescence (PCL), and ferric reducing ability of plasma (FRAP) [73]. The purpose of all previous tests is to measure antioxidant activity. However, factors such as the structure of the analyzed molecule will indicate which tests are more suitable for each occasion.

Thus, to suggest a preventing property against oxidative stress in the body is important for a beer to be enriched with antioxidant compounds [72]. This potential can be evaluated by several colorimetric tests that seek to quantify antioxidant compounds. Colorimetric methods are well evaluated and used for their simplicity and performance. The most commonly applied to beverages are Trolox equivalent antioxidant capacity (TEAC), total peroxyl radical trapping antioxidant parameter (TRAP), 2,2-diphenyl-1-picrylhydrazyl (DPPH), *N*,*N*-dimethyl-*p*-phenylenediamine (DMPD), photochemiluminescence (PCL), and ferric reducing ability of plasma (FRAP) [73]. The purpose of all previous tests is to measure antioxidant activity. However, factors such as the structure of the analyzed molecule will indicate which tests are more suitable for each occasion.

Table 2 shows the data for antioxidant activity of the functional beers, showing which test was employed and the activity obtained. It can be seen in Table 2 that the antioxidant tests used were DPPH, FRAP, ABTS, and ORAC. Following the previously mentioned results from the TPC, the addition of fruits in the boiling step offered the highest observed increases in the antioxidant capacity of beers. Furthermore, when compared for the same fruit, the results of the antioxidant activity of the boiling steps were superior to those of fermentation, maturation, and before packaging. 

These results are in line with the findings for the total phenolics. Such a correlation between a higher TPC and higher antioxidant activity is expected as phenolics are a known class of antioxidant compounds [53]. Lastly, except for the porter beer with *P. lucida,* the plant extracts and propolis showed low increases in antioxidant activity, which brings attention to the evaluation of the bioactive content of the added adjuncts.

From Table 2 it becomes clear that, in general, an increase in beer’s antioxidant activity occurs after the addition of adjuncts. Although this finding is positive and highlighted throughout the results and discussion of the articles, to infer that both the total concentration of antioxidants and their antioxidant activity may be available to the organism upon consumption is to overestimate it [74]. As stated by Granato et al., the measurement of TPC and antioxidant activity by colorimetric assays, although valuable, especially in a trial for adjunct selection, are not enough to establish a bioactive value to the product [63]. The in vitro colorimetric assays are restricted to analyzing beer samples and were not concerned with the compound’s bioavailability (Table 2). Thus, the actual in vivo assays, such as cell lineages and bioavailability, must be included in studies addressing this field of study [75].

The effects of gastrointestinal digestion can be seen in simulated digestion [76], such as Koehnlein et al.). The authors make a comparison between the aqueous extraction and in vitro digestion of 36 popular foods in Brazil concerning their phenolic content and antioxidant activity [76]. In vitro digestion simulated the different stages of the gastrointestinal tract. It was found that for beverages tested, including a commercial Pilsen beer, a reduction in TPC and antioxidant activity was found after the digestion process. This reduction is attributed to the bio-accessible nature of the bioactive compounds in these beverages, which makes them more susceptible to changes during the digestion process.

It is important to note a lack of bioavailability data in the bibliography, including functional beers. Other studies that lack data are those that focus on the absorption of phenolic compounds. This information helps to understand how the compound is distributed, metabolized, and absorbed, ultimately determining the beverage as bioactive.

## 4. Volatile Compounds and Their Influence on the Sensory Profile of a Beer

The presence of adjuncts in beer adds value by increasing bioactive compounds with antioxidant potential and improving the sensory characteristics. The sensory profile of a traditional beer depends on the raw materials used and their interactions throughout the production process [6]. When an adjunct is added, it became another variable that can influence the product’s sensory profile. In practical terms, phenolic compounds from the raw materials impart astringency and bitterness to beer. However, the consumer’s greatest variety of sensations comes from volatile compounds extracted from the ingredients and those chemically modified by the manufacturing process condition. They represent aliphatic and aromatic alcohols, esters, organic acids, aldehydes, carbonyl compounds, and terpenes [77]. On the one hand, is sought those volatile compounds that are harmonic with the produced beer remain or be formed in the beverage. On the other hand, unpleasant compounds known as off-flavors should be eliminated or reduced throughout production, and if possible, not generated during storage.

Sensory descriptions of beer are elegantly presented and organized in flavor wheels. This way of cataloging and ordering several sensory terminologies was first proposed by Meilgaard et al. in 1979 [78]. The published study was a partnership between the European brewery Convention (EBC), the American Society of Brewing Chemists, and the Master Brewer’s Association of the Americas. The developed system includes unique, objective, and standardized terms that give rise to fourteen main classes that have further secondary divisions to cover specific flavor notes found in the beverage. Later, Langstaff and Lewis contributed to updating the previous wheel with adaptations in the mouthfeel to make this class more specific and detailed [79]. It is noteworthy that updates were created over time as the terminologies are dynamic and need to be revised as novel compounds are identified, and new beers developed. To be classified according to the technical vocabulary, the compound needs to be sensorily identified, which is only possible if the compound concentration in the beer is higher than the sensitivity threshold of the human senses (the compound flavor threshold), making the compound flavor active. The method used to screening a matrix, such as beer, find flavor active molecules, and give descriptive sensorial notes is the gas chromatographic-olfactometry (GC-O). In this technique, after chromatographic separation, the sample is simultaneously led through an analytical detector (such as FID or MS) and a sniff port where a trained individual can detect and describe the compound flavor [6]. In general, compounds are divided into four classes (primary, secondary, tertiary, and background) depending on the ratio of the compound concentration in relation to their sensitivity threshold [80].

Therefore, analysis of volatile compounds in beers is imperative to understand how adjuncts may influence the beers’ aromatic profile. The standard analysis for detecting and identifying volatile compounds in beer is gas chromatography coupled with mass spectrometry (GC-MS) [6]. Furthermore, as direct injection of beer in the GC can lead to contamination of the equipment, it is common to sample the beer headspace (HS), where solid-phase microextraction (SPME) techniques are the most used in functional beer studies. Sampling the beer headspace is highly effective in extracting the volatile profile of a complex matrix such as beer, and the use of SPME reduces the interference of non-volatile compounds. Furthermore, SPME requires minimal samples preparation and avoids using organic solvents, following the trend of green chemistry [81].

Deng et al., used HS-SPME-GC-MS to evaluate acetaldehyde, diacetyl, higher alcohols, and esters content in an omija beer [11]. The addition of omija to wort boiling increased acetaldehyde and esters, while no dfferences in higher alcohols was found. Esters mainly contribute to the fruity aroma of a beer and, in general, are perceived as positive. It is worth highlighting that isoamyl acetate is known to mask the perception of off-flavors in beer [16]. Therefore, it can be inferred that the sensory stability of the omija beer is higher than the control. The addition of omija fruit before and after fermentation did not cause a change in the volatile profile of the resulting beers compared to the control. Furthermore, the beer produced with the addition of omija during boiling increased sourness, as perceived in the sensory analysis panel. However, the omija beer had the same acceptability as the control beer.

In their work, Xu et al., used HS-SPME-GC-MS to evaluate the volatiles resulting from the addition of okra during wort boiling [17]. Among esters, alcohols acids, aldehydes, terpenes, and ketones, the authors identified a total of 57 volatile compounds. In general, after adding okra, a lower higher alcohols level and an increase in esters was found for beers enriched with fresh and dried okra. This represents a change in flavor from a more pungent aspect, related to the main higher alcohol, isoamyl alcohol, to a fruitier flavor related to esters such as ethyl acetate. Besides, the okra beer contained caryophyllene, suggesting a new aroma related to the addition of the adjunct. Overall, the addition of okra was beneficial for the beer’s aromatic profile, increasing the content of esters without adding any potential off-flavors. As for the sensory evaluation, the okra beer was perceived with higher turbidity, color intensity, and grassy-like odor, while less bitter and sour than the control.

Zapata et al., added quince pieces to amber ale beers during a 10-day maturation process [52]. The authors analyzed the volatile profile of the quince beer with HS-SPME and found 34 distinct volatiles. By relative quantification, it was found that quince beers were richer in esters with fruity and floral notes. No off-flavor compound was detected resulting from the addition of quince. The sensory analysis of beer was composed of 24 flavor and visual attributes raised by the trained panelists. The quince beer color had better evaluation than the control. Furthermore, the hop, floral, fruity, bitter notes, and the beer body were better evaluated for quince beer. On the other hand, the beer caramel notes were deemed inferior. The association of other volatiles has likely decreased the caramel note naturally found in American amber ale beers used as a manufacturing base for this study.

Adadi et al., analyzed a sea buckthorn beer by direct injection of the sample in the GC-MS. The authors found 32 volatile compounds for the sea buckthorn beer, consisting of 16 esters, 10 organic acids, and six alcohols [38]. The study did not present a comparison between the novel and a control beer. Therefore, it is unclear which differences in the beer volatile profile were a result of the adjunct addition. Volatile acids have been indicated as potential influences of both beer pH and flavor, although esters are the major actors in flavor [13]. The results from the sensory analysis showed that the sea buckthorn beer was well accepted, with an average, with good color, aroma, and mouthfeel scores.

Sensory evaluation of the studied functional beer was not unanimously found in the scientific literature (Table 1). Except for the Nardini and Garaguso work, which evaluated commercially available beers, the lack of sensory evaluation data is worrisome. Although the use of plant extracts, mushrooms, and propolis can be very attractive due to the tremendous functional potential of these adjuncts, care should be taken to produce a sensory attractive beverage. It should be highlighted that the drinks’ color is one factor that most affects the consumer’s purchase decision since the visual experience influences the expectation of other organoleptic properties [82]. Corroborating this notion, Pappalardo and Lusk indicated that, although consumers would even pay higher prices for functional products, the willingness to pay can be affected by the product quality [83].

## 5. Conclusions

Functional beers are a viable and little-explored option within food science to provide human health benefits. The production of these non-traditional beers requires a combination of the added adjunct and an addition step. On the one hand, the addition of fruits to the boiling steps proved to be attractive as the extraction of phenolic and bioactive compounds is enhanced in this step, which is reflected by the high total phenolic content and antioxidant potential of the beers produced. Furthermore, higher extraction of volatiles and was obtained in beer with fruits added to the boiling step, which led to good sensory acceptability. The addition of plant extracts was mainly found in maturation steps and resulted in general in low increases in antioxidant and phenolic content of beers. Furthermore, the studies showed it to be possible for bioactive compounds’ obtention without neglecting the sensory analysis of the product as most of the designed beer obtained equal or superior scores in sensory analysis.

Although the transfer of bioactive substances from the adjuncts to the final beer is proven, the direct benefits to human health are substantial. From the technological perspective, state-of-the-art techniques such as LC-MS must be employed to profile the bioactive compounds incorporated into the beverage. Furthermore, it is necessary to establish a circular work, addressing how and which adjunct will be added taking into account the physicochemical, sensory, and physiological terms as bioavailability. Then, the functional claim on these beverages will be completely supported. However, as a new frontier in beer science, the field of functional beers offers a valuable and rich path for new studies.

## Figures and Tables

**Table 1:** Detailed compilation of studies regarding the production of functional beers and their total phenolic content.

Adjunct	Classification	Production Mechanism	Addition Mechanism	Conventional Beer TPC (mg GAE/L)	Enriched Beer TPC (mg GAE/L)	Sensory Evaluation	Reference
Okra	fruit	wort boiling	dried powder	-	-	yes	[17]
			pulp				
Omija fruit	fruit	wort boiling	crushed and dried	519	606	yes	[11]
		fermentation			575		
		maturation			568		
Goji berry	fruit	wort boiling	ground and dried	335	609	yes	[22]
			whole and dried		623		
		fermentation			373		
		maturation			415		
		before packaging	ground and dried		357		
Peach	fruit	fermentation	pieces	500	506–618	yes	[50]
Persimmon	fruit	fermentation	pieces	507	595–714	yes	[27]
Cherry	fruit	fermentation	pieces	321–482	747–767	no	[29]
Raspberry					465–536		
Peach					510		
Apricot					454		
Grape					631		
Plum					598		
Orange					639		
Apple					399		
Green pepper	fruit	fermentation	pieces	723	1009–1321	yes	[61]
Bush passion fruit	fruit	fermentation	pulp	-	-	no	[32]
Banana	fruit	fermentation	pulp	-	-	no	[34]
Cocoa	fruit	fermentation	pulp	-	-	yes	[31]
Quince	fruit	maturation	pieces	13.47 *	15.90–17.55 *	yes	[52]
Sea buckthorn berry	fruit	maturation	pieces	-	-	yes	[38]
Pineapple	fruit	maturation	pulp	-	-	no	[62]
*P. lucida*	plant	maturation	dried leaves	413	480–800	no	[37]
Propolis	resinous mixture	maturation	extract	242	253–306	no	[40]
*Melissae folium*	plant	after packaging	extract	280	363	yes	[43]
*T. herba*	plant				384		
*U. radix*	plant				317		
*J. fructus*	fruit				365		
*L. strobuli*	plant				316		
*G. lucidum* L.	mushroom	after packaging	extract	-	-	yes	[45]
*G. lucidum* L.	mushroom	after packaging	extract	<300	<900	no	[44]
			microencapsulated		<300		
*C. sinensis* L.	plant		extract	<300	<600		
			microencapsulated		<1200		

* indicates the only measure in a different unit, (mg pyrogallol/100 g).

**Table 2:** Comparison of adjunct used, production stage of insertion, and resultant antioxidant value.

Adjunct	Classification	Production Stage	Conventional Beer—DPPH Assay	Enriched Beer—DPPH Assay	Conventional Beer—FRAP Assay	Enriched Beer—FRAP Assay	Conventional Beer—ABTS Assay (mM TE)	Enriched Beer—ABTS Assay (mM TE)	Conventional Beer—ORAC Assay (mM TE)	Enriched Beer—ORAC Assay (mM TE)	Reference
omija fruit	fruit	wort boiling	0.88 mM TE(1)	2.02 mM TE(1)	1.79 mM Fe+2(1)	3.01 mM Fe+2(1)	-	-	-	-	[11]
		fermentation		1.68 mM TE(1)		2.40 mM Fe+2(1)	-	-	-	-	
		maturation		0.96 mM TE(1)		1.86 mM Fe+2(1)	-	-	-	-	
goji berry	fruit	wort boiling	-	-	-	-	2.26	3.82	8.87	16.81	[22]
				-		-		3.70		16.84	
		fermentation		-		-		2.87		15.04	
		maturation		-		-		2.95		13.13	
		before packaging		-		-		2.40		10.03	
peach	fruit	fermentation	86.11% inhibition	87.53–88.90% inhibition	-	-	-	-	-	-	[50]
persimmon	fruit	fermentation	80.12% inhibition	85.31–91.00% inhibition	-	-	-	-	-	-	[27]
cherry	fruit	fermentation	-	-	2.80–4.39 Fe_2_SO_4_ eq. mM	8.55–9.76 Fe_2_SO_4_ eq. mM	1.29–2.03	3.41–3.53	-	-	[29]
raspberry				-		4.87–5.71 Fe_2_SO_4_ eq. mM		1.98–2.35		-	
peach				-		4.56 Fe_2_SO_4_ eq. mM		1.86		-	
apricot				-		4.20 Fe_2_SO_4_ eq. mM		1.66		-	[29]
grape				-		6.85 Fe_2_SO_4_ eq. mM		2.81		-	
plum				-		5.66 Fe_2_SO_4_ eq. mM		1.93		-	
orange				-		5.65 Fe_2_SO_4_ eq. mM		1.67		-	
apple				-		3.08 Fe_2_SO_4_ eq. mM		1.62		-	
*P. lucida*	plant	maturation	-	-	1.88 mM TE	2.17–5.46 mM TE	1.15	1.38–3.34	7.86	10.14–30.58	[37]
Propolis	resinous mixture	maturation	0.53 mM TE	0.49–0.57 mM TE	1.41 mM TE	1.55–1.89 mM TE	0,62	0.68–0.80	-	-	[40]
*M. folium*	plant	after packaging	2.54 mM TE	3.05 mM TE	4.15 mM TE	4.51 mM TE	-	-	-	-	[43]
*T. herba*	plant			3.72 mM TE		4.71 mM TE		-		-	
*U. radix*	plant			2.85 mM TE		4.25 mM TE		-		-	
*J. fructus*	fruit			3.14 mM TE		4.55 mM TE		-		-	
*L. strobuli*	plant			2.83 mM TE		4.27 mM TE		-		-	
